# Supplementary material for: Interstitial cystitis/bladder pain syndrome patient is associated with subsequent increased risks of outpatient visits and hospitalizations: A population-based study
Source: PLoS One. 2021 Sep 7;16(9):e0256800. doi: 10.1371/journal.pone.0256800 (PMC8423233; doi:10.1371/journal.pone.0256800)
Supplement: Attachment — Submitted filename: ICBPS_to_OPD_response_2021.04.14 (finished).docx [file pone.0256800.s001.docx]

Response to academic editor and reviewers:

We would appreciate our deep thanks for the Academic Editor, and Reviewers’ sophisticated reading on our manuscript, and very instructive and important comments and suggestions. Below were our responses in point-to-point pattern. Enclosed please find our response letter, marked version and clean version. We hope our devotion and revision on the manuscript can merit the acceptance and publication in high impact journal, PLOS ONE.

PONE-D-21-02197
Interstitial cystitis/ Bladder pain syndrome patients are associated with subsequent increased risks of out-patient visits and hospitalizations: a population-based study

==============================
ACADEMIC EDITOR:

Q: Based on the comments of the reviewers, I ask that you include your changes very carefully in the revision of the manuscript. Especially the discussion should be more focused and better organized. But also: Make it much clearer how your findings can influence (improve) of the quality of care for people with this syndrome.

Response: Thank you for your comments and suggestions. We have revised accordingly. We have revised and organized our discussion to focus on our findings and delete some paragraphs which were irrelevant to our study.

【p.27, line 260-270】 We deleted the paragraph related to colo-rectal surgery, since many of the relationship between bladder condition and relationship about irritable bowel syndrome, etc are still hypothetic, which is out of the scope of our study. Again, we appreciate the important point of view.

【p.30, line 325-330】【p.27, line 268-270】 We have deleted the paragraph about pathogenesis, e.g. GAG defect and other mechanism, because of irrelevant information, unrelated to our data. We also delete the theory about “ cross sensitization” since our data could not offer evidence to support it.

Also, to follow your instructive suggestions, we tried to make more clear how this study can influence the quality of care. “Our study and other reports may remind physicians to pay attention to patients with IC/BPS in a broader view, in medical, surgical and mental illness. Adequate recognition and possible recommendation or referral for IC/BPS patients not only provide better healthcare quality, even decrease catastrophic consequences”【p.25, line 222-225】. And we emphasized that our findings broaden our understanding about IC/BPS, from organ-centered to multiple system concepts in conclusion “This study also provides a broader understanding of IC/PBS within multiple and overlapping systems. These findings and explanations are emphasized that patient with IC/PBS should be paid more attention due to systemic complex disease and higher medical service.”【p.32, line 365-369】

Q: You also write that the "pathophysiology is unknown." In a large number of people (without Hunner's lesion) the syndrome is not very distinct from overactive bladder syndrome. Can it be determined whether it is excluded in some of the people that specific dysfunction plays a role in the syndrome where the patients refer their signs and symptoms to as pain? And whether you have any information whether the pain has been objectively shown to be related to the lower urinary tract, more specifically, filling (stretching) of the bladder? (are these in local (national) guidelines? as is 'exclusion of other causes', in the international guidelines?)

Response: Thanks for your comments. Since this is a study based on claim data, the inclusion and exclusion criteria of IC/BPS are up to individual physician’s clinical opinions. However, this study included IC/BPS patients who received bladder instillation treatment, which requires a strict pre-review audit system. There are definite rules in payment regulations of National Health Insurance [Ref National Health Insurance]. “Its provision included: the presence of glomerulation or Hunner’s lesions under cystoscopy after hydrodistention test, voiding diary, negative urine cytology and urine analysis “ [p.6 Material and Methods session, Assembly of study participants]. The above guideline is according to the guideline of Taiwan Urology Association, in line with AUA [Ref TUA guideline] [Ref AUA guideline]

Ref: Taiwan National Health Insurance, https://www.nhi.gov.tw/Content_List.aspx?n=197F438368327FE0&topn=5FE8C9FEAE863B46

Ref: TUA guideline, https://online.fliphtml5.com/thpbe/cljw/#p=1

Ref: AUA guideline, <https://www.auanet.org/guidelines/interstitial-cystitis-(ic/bps)-guideline>

==============================


Reviewers' comments:

Reviewer's Responses to Questions

**Comments to the Author**

1. Is the manuscript technically sound, and do the data support the conclusions?

The manuscript must describe a technically sound piece of scientific research with data that supports the conclusions. Experiments must have been conducted rigorously, with appropriate controls, replication, and sample sizes. The conclusions must be drawn appropriately based on the data presented.

Reviewer #1: Yes

Reviewer #2: Yes

Response: Thanks.

2. Has the statistical analysis been performed appropriately and rigorously?

 Reviewer #1: Yes

Reviewer #2: Yes

Response: Thanks.

3. Have the authors made all data underlying the findings in their manuscript fully available?

The [PLOS Data policy](http://www.plosone.org/static/policies.action#sharing) requires authors to make all data underlying the findings described in their manuscript fully available without restriction, with rare exception (please refer to the Data Availability Statement in the manuscript PDF file). The data should be provided as part of the manuscript or its supporting information, or deposited to a public repository. For example, in addition to summary statistics, the data points behind means, medians and variance measures should be available. If there are restrictions on publicly sharing data—e.g. participant privacy or use of data from a third party—those must be specified.

Reviewer #1: Yes

Reviewer #2: Yes

Response: Thanks.

4. Is the manuscript presented in an intelligible fashion and written in standard English?

PLOS ONE does not copyedit accepted manuscripts, so the language in submitted articles must be clear, correct, and unambiguous. Any typographical or grammatical errors should be corrected at revision, so please note any specific errors here.

Reviewer #1: Yes

Reviewer #2: No

Response: After the manuscript revision, we have made substantial English editing.

5. Review Comments to the Author

Reviewer #1: The manuscript is well written overall. While the data is clearly presented, and it is clear from the manuscript that patients with IC/BPS have higher rates of outpatient visits and higher rates of hospitalization, some of the discussion is conjecture, not based on the data.
The manuscript would be stronger if it simply focused on the finding of higher visits/costs/resource utilization, and did not stray into physiology such as "cross organ sensitization" between the bladder and rectum. While this may be true (i believe it is), it is neither related to nor supported by the data presented.

Response to Reviewer 1: We agreed the Reviewer’s comment that some discussion opinions were conjecture, not based on the data. We have revised and organized our discussion to more focus on our findings. Including:

【p.27, line 260-270】 We deleted the paragraph related to colo-rectal surgery, since many of the relationship between bladder condition and relationship about irritable bowel syndrome, etc are still hypothetic, which is out of the scope of our study. Again, we appreciate the important point of view.

【p.30, line 325-330】【p.27, line 268-270】 We have deleted the paragraph about pathogenesis, e.g. GAG defect and other mechanism, because of irrelevant information, unrelated to our data. We also delete the theory about “ cross sensitization” since our data could not offer evidence to support it.

And we also did other revisions.

Besides, we tried to pay more attention to influence of the findings. In 2nd paragraph of discussion 【p.24-25】”Our study and other reports may remind physicians to pay attention to patients with IC/BPS in a broader view, in medical, surgical and mental illness. Adequate recognition and possible recommendation or referral for IC/BPS patients not only provide better healthcare quality, even decrease catastrophic consequences.

Q: Furthermore, the concept of hunner's ulcer as "ic" and non-hunner's ulcer as BPS is interesting, and likely true. The presence of hunner's ulcer is likely a different phenotype and different disease process, but it has virtually nothing to do with the finding of increased utilization, visits, hospitalizations, and should be omitted from the manuscript.

Response: Thanks for your suggestions and comments. In this study, we did not try to discuss on the issue of Hunner-type, or non-Hunner-type, because data inavailability. There are different diagnostic criteria and classifications in IC/BPS. As our response to editor mentioned above, our claim data was collected from NHIRD with inherent claim data characteristics and limitations. We only recruited IC/BPS patients who received bladder instillation treatment after strict pre-review audit system. Therefore our IC/BPS cases only confined to Hunner type (or with glomerulation) due to claim data characteristics. Whether our findings can be deductive to non-Hunner type IC/BPS remains inclusive. It is out of the scope of our study.

Reviewer #2: This is a study of the rate of out-patient visits and hospitalization in a group of patients with a diagnosis of IC/BPS compared to age matched controls. Patients with IC/BPS had more hospitalizations

Q1. The review criteria for getting the hyaluronate includes the presence of glomerulations or Hunner’s lesions under cystoscopy after hydrodistension- this criteria for glomerulations is no longer used and was part of the old 1988 NIH guidelines. Current guidelines only use symptoms (ref 1 in the paper). Can you comment on how this difference in diagnosis could influence the patient population being studied?

Response: We agreed with the Reviewer’s comment that the finding of glomerulations is no longer used in modern clinical practice. However, due to claim data characteristics, we only recruited IC/BPS patients who received bladder instillation treatment, which requires a strict pre-review audit system. “Therefore our IC/BPS cases only confined to Hunner type (or with glomerulation). Whether this difference between symptom-based and cystoscopic-based diagnosis could influence the patient population remain inclusive according to our data setting. This might need case control trial to compare these two diagnostic criteria setting. It is also out of the scope of our study.”【p. 32, line 356-360】Again, we thank for reviewer’s important and instructive comments, we revised it in the limitation.

Q2. Was there a gender difference in the increase in Urology visits compared to controls? In any of the other non GYN visits?

Response: Yes, we found higher risk of male IC/BPS patients than female in Urology visits and also hospitalization. The rate of Urology visit is female (1.07) and male (2.33) (per one person-year) with adjusted IRR were 0.42 (95% CI: 0.40-0.45). The rate of Urology admission is female (23.85) and male (72.94)(no. per 1000 person-year) with adjusted IRR were 0.34 (95% CI: 0.29-0.40).We had added this data to p.12 and p18.

Q3. Top of page 26, you cite reference 19 for increased rate of cancer in patients with IC/BPS. This is the incorrect reference – should be Wu MP, Luo HL, Weng SF, Ho CH, Chancellor MB, Chuang YC Risk of Urinary Tract Carcinoma among Subjects with Bladder Pain Syndrome/Interstitial Cystitis: A Nationwide Population-Based Study. BioMed Research International. 2018:7495081, 2018.
Response: We apologize for the mistake. We have corrected the reference number into our text. We appreciated the Reviewer’s sophisticated reading on the manuscript.

4. 4. Bottom of page 27- you postulate that the patients go to orthopedics and neurosurgery more due to risk of falls. You reference two articles that deal with the association of LUTS and specifically urgency in older women and men. However 73% of your cohort is under the age of 60. You did not break out the age in table 4.

Response: We appreciate the Reviewer’s pointing our mistake. We agreed that the explanation is hypothetic. We further cited a more related reference to explain the possible relationship. Moon SJ et al indicated that higher falling rate in females aged 40 and older due to urgency and LUTS. Also Kurita N et al. found that higher OAB symptoms were associated to high fall risk from 40 year-old and older. [Ref 24 and 25. revision]. These studies showed that high fall risk not only in elderly but also age older than 40.

24	Moon SJ, Kim YT, Lee TY, Moon H, Kim MJ, Kim SA, et al. The influence of an overactive bladder on falling: a study of females aged 40 and older in the community. Int Neurourol J. 2011; 15(1): 41–7.

25	Kurita N, Yamazaki S, Fukumori N, Otoshi K, Otani K, Sekiguchi M, et al. Overactive bladder symptom severity is associated with falls in community-dwelling adults: LOHAS study. BMJ Open. 2013; 3(5):e002413
