# Supplementary material for: Interstitial cystitis/bladder pain syndrome patient is associated with subsequent increased risks of outpatient visits and hospitalizations: A population-based study
Source: PLoS One. 2021 Sep 7;16(9):e0256800. doi: 10.1371/journal.pone.0256800 (PMC8423233; doi:10.1371/journal.pone.0256800)
Supplement: Attachment — Submitted filename: ICPBS 2021.07.29 (revision2)(answer to reviewer).docx [file pone.0256800.s002.docx]

Response to academic editor and reviewers:

We would appreciate our deep thanks for the Academic Editor, and Reviewers’ sophisticated reading on our manuscript, and very instructive and important comments and suggestions. Below were our responses in point-to-point pattern. Also we revised out manuscript under reviewers’ suggestions to improve this paper. Enclosed please find our response letter, marked version and clean version. We hope our devotion and revision on the manuscript can merit the acceptance and publication in high impact journal, PLOS ONE.

Thanks for reviewer 2 and here are responses to reviewer 3:

1) Methods Section, Lines 104-109. IC/BPS cases are followed from “index date of first registration”, not date of diagnosis. It is unclear how long patients are followed prior to date of initial diagnosis, nor how the inclusion of total time followed impacts the analysis. Or if I am mis-reading this, is index date is equivalent to date of diagnosis? Please add more clarity to describe this.

Ans: Thanks for your question. We apologize for the lack of clarity. We have tried to define and revise the sentence. The index date is first registration with the diagnostic code of IC/BPS (ICD 9 code 595.1). Later on, the cases who received bladder instillation as mentioned and at least 3 outpatient visits with the same diagnosis at OPD visit were included. .

We add more information in Lines 110-112.

2) Methods, Lines 128-134: In the analysis of impact of gender or age differences, were these calculated as interaction terms in the model?

Ans: Thanks for your comment. Due to the age and gender had been matched, the interaction terms of age and gender did not be included in the model for avoiding the over adjustments.

3) Methods, Lines 131-133: Please indicate how missing data on covariates was handled. Was missing data coded, or were cases excluded with missing data?

Ans: Thanks for your question. We apologize for this mistyped error. We want to tell “Some other potential confounders were NOT available due to claim data characters, such as education…”. We correct it in Line 132. Thanks for your careful reading again.

4) Table 1: p-values cannot be 1.0. Recommend change to p>0.9999 for the notation.

Ans: Thanks for reviewer’s suggestion. The p-value had been modified, accordingly. (We correct in Table 1)

5) Results: Lines 152-153: Regarding the gender difference in urology rates, please clarify if these are the overall gender rates for Urology. Or are these the specific for just the IC/BPS group? Clarify the description of the IRR rate that this is female to male “...adjusted IRR rate for females to males was 0.42...”

Ans: Thanks for review’s question and suggestion. Yes, our description was not so clear. The gender difference in urology rates is specific for patients with IC/PBS. And the adjust IRR is female to male. We will make more clear description of gender difference in Lines 153-155.

6) Results, Lines 165-166: Regarding the differences observed across ages, if these rates are increasing, please provide the results of the statistical tests with the appropriate p-value for trend. Were each pairwise contrast of Group 1 to 2 and Group 2 to 3 significantly different?

Ans: Thanks for the reviewer’s comment. The trend test of outpatient visits rates for patients with IC/PBS following the increased age groups had presented as the below table. The trend test of each pairwise contrast groups was also presented. These p-values was added in the below of Table3 (Line 182)

| p-value for trend | Overall | 40≦ vs. 40-60 | 40-60 vs. ≧60 | 40≦ vs. ≧60 |
| --- | --- | --- | --- | --- |
| Patients with IC/PBS |  |  |  |  |
| Outpatient visits | <.0001 | <.0001 | <.0001 | <.0001 |

7) Results, Lines 194-199: Please provide p-values to support these statement of significant differences between age groups.

Ans: Thanks for the reviewer’s comment. The differences between age groups all show p-value<.0001. These p-values were added in the manuscript to explain the statement of differences between age groups. The paragraph was modified as below. (Line 200-207)

“Significant higher IRR in younger age comparing to older age group in urology department (IRR: 11.33 vs. 5.55 and 5.38, p-value of age groups was p<.0001). This result is similar in most departments, including psychiatry,

orthopedics, neurology and neurosurgery. The IRR of hospitalization in gynecology is higher IRR in oldest group (less than 40 years: 1.45 vs. 40 to 60 years: 2.29 vs. older than 60 years: 5.70, p-value of age groups was p<.0001). ”

8) The manuscript would benefit from a detailed editing by a native English speaker.

Ans: Thanks for review’s suggestion. Due to the authors are not native English speakers, we sent the manuscript to international editing company for help. This new version of manuscript had been edited by the editors from Enago.
